# Micronutrient Deficiency During Pregnancy After Bariatric Surgery: The Role of Micronutrient Supplements and Dietary Intake

**DOI:** 10.1007/s11695-026-08576-7

**Published:** 2026-03-25

**Authors:** Taylor Guthrie, Jade Eccles-Smith, Sandra Lee, Alka Kothari, William Pinzon Perez, Helen Truby, Susan de Jersey

**Affiliations:** 1 Faculty of Health, Medicine and Behavioral Sciences, University of Queensland, Brisbane, Australia; 2 Dietetics and Foodservices, Royal Brisbane and Women’s Hospital, Brisbane, Australia; 3 Department of Obstetric Medicine and Endocrinology, Royal Brisbane and Women’s Hospital, Brisbane, Australia; 4 Mater Research Institute, University of Queensland, Brisbane, Australia; 5 Maternity Services, Caboolture Hospital, Caboolture, Australia; 6 Department of Obstetrics and Gynecology, Redcliffe Hospital, Redcliffe, Australia; 7 Queensland Cyber Infrastructure Foundation Ltd, Brisbane, Australia

**Keywords:** Bariatric surgery, pregnancy, micronutrient deficiency, micronutrient supplementation, dietary intake

## Abstract

**Background:**

Pregnancy markedly increases micronutrient demands; however bariatric surgery can compromise dietary micronutrient intake and absorption. International Consensus Guidelines recommend additional micronutrient supplementation during pregnancy following bariatric surgery to prevent deficiencies; however, their efficacy is unknown. This study examined whether women met recommended micronutrient supplementation during pregnancy and explored the relationship between micronutrient intake and deficiency during pregnancy.

**Methods:**

Pregnant women who had bariatric surgery prior to conception, aged 18–45 years were recruited prospectively before 23 weeks’ gestation and followed until birth. Multiple pass 24-hour diet recalls assessed dietary micronutrient intake. Micronutrient supplementation and adherence was reported pre-pregnancy, at enrollment, and at 28- and 36-weeks gestation. Maternal blood values were obtained from medical records after birth. Logistic regression examined the role of micronutrient intake in the development of biochemical iron, zinc, copper, selenium, folate, and vitamins A, B12, D, and E deficiencies.

**Results:**

Sixty-nine women, aged 31 ± 4.8 years, participated. Multivitamin use increased from 38/69 (55%) pre-pregnancy to 55/69 (80%) at enrolment. The proportion meeting supplementation recommendations was low across all micronutrients, ranging from 38/69 (59%) for selenium to 0/69 for vitamin A. Most participants (56/69, 81%) developed a micronutrient deficiency during pregnancy, most commonly iron (49/69, 72%,) and vitamin B12 (38/69, 54%) followed by vitamin A (21/69, 30%) and vitamin D (21/69, 30%). Though micronutrient intake was correlated with micronutrient concentrations during pregnancy, the study was underpowered to detect predictors of deficiency.

**Conclusion:**

Bariatric surgery recipients infrequently met supplement recommendations during pregnancy and the relationship between micronutrient intake and deficiency was not clear. Iron, vitamin A, B12 and D deficiencies were common and warrant routine monitoring in antenatal care post-bariatric surgery.

**Supplementary Information:**

The online version contains supplementary material available at 10.1007/s11695-026-08576-7.

## Keypoints

International Consensus Guidelines recommend additional micronutrient supplementation for pregnant women with a history of bariatric surgery.

This study found that although most women took multivitamin multimineral supplements during pregnancy, these seldom met the doses recommended in the International Consensus Guidelines.

The majority of participants (81%, 56/69) experienced at least one micronutrient deficiency during pregnancy, most commonly iron (49/69, 72%) and vitamin B12 (38/69, 54%).

Micronutrient supplementation and dietary intake influenced maternal serum concentrations of vitamin D, E, folate and zinc, but did not demonstrate a clear impact on the odds of deficiency.

**Supplementary Information:**

The online version contains supplementary material available at 10.1007/s11695-026-08576-7.

## Introduction

Bariatric surgery achieves significant weight loss and improves metabolic health [1]. Despite this, an undesirable complication is micronutrient deficiency, likely attributable to small dietary portion sizes coupled with compromised absorption of micronutrients [2]. Vitamins A, B12, D, E, calcium, folate, thiamine, iron, zinc, and copper deficiencies have been reported in adults post-operatively [1, 3]. To attenuate this, British and American guidelines recommend lifelong vitamin and mineral supplementation following bariatric surgery [1, 4]. Women of childbearing age now make up a large and growing number of adults who are undergoing bariatric procedures [5, 6], potentially motivated by evidence highlighting the benefits of surgery-induced weight loss on fertility [7, 8] and some pregnancy outcomes [9–12]. As pregnancy is associated with a marked increase in micronutrient requirements, pregnant women post-bariatric surgery may be more vulnerable to deficiency. A recent systematic review reported micronutrient deficiency in up to 90% of pregnant women with a history of bariatric surgery [13]. Micronutrient deficiency during pregnancy is associated with several adverse perinatal outcomes such as congenital anomalies, gestational diabetes mellitus, pregnancy-induced hypertension and pre-eclampsia, preterm birth, and small for gestational age or growth restricted infants [14–18]. Maternal depletion of micronutrients during pregnancy may result in reduced neonatal stores, predisposing infants to deficiency in early life [19]. Breastfed infants also rely on adequate concentrations of vitamins A, B12, D, folate and zinc in breast milk to maintain their micronutrient status [20]. It is therefore critical for maternal and offspring health outcomes to attenuate the risk of micronutrient deficiency during a pregnancy following bariatric surgery.

**Fig. 1:**
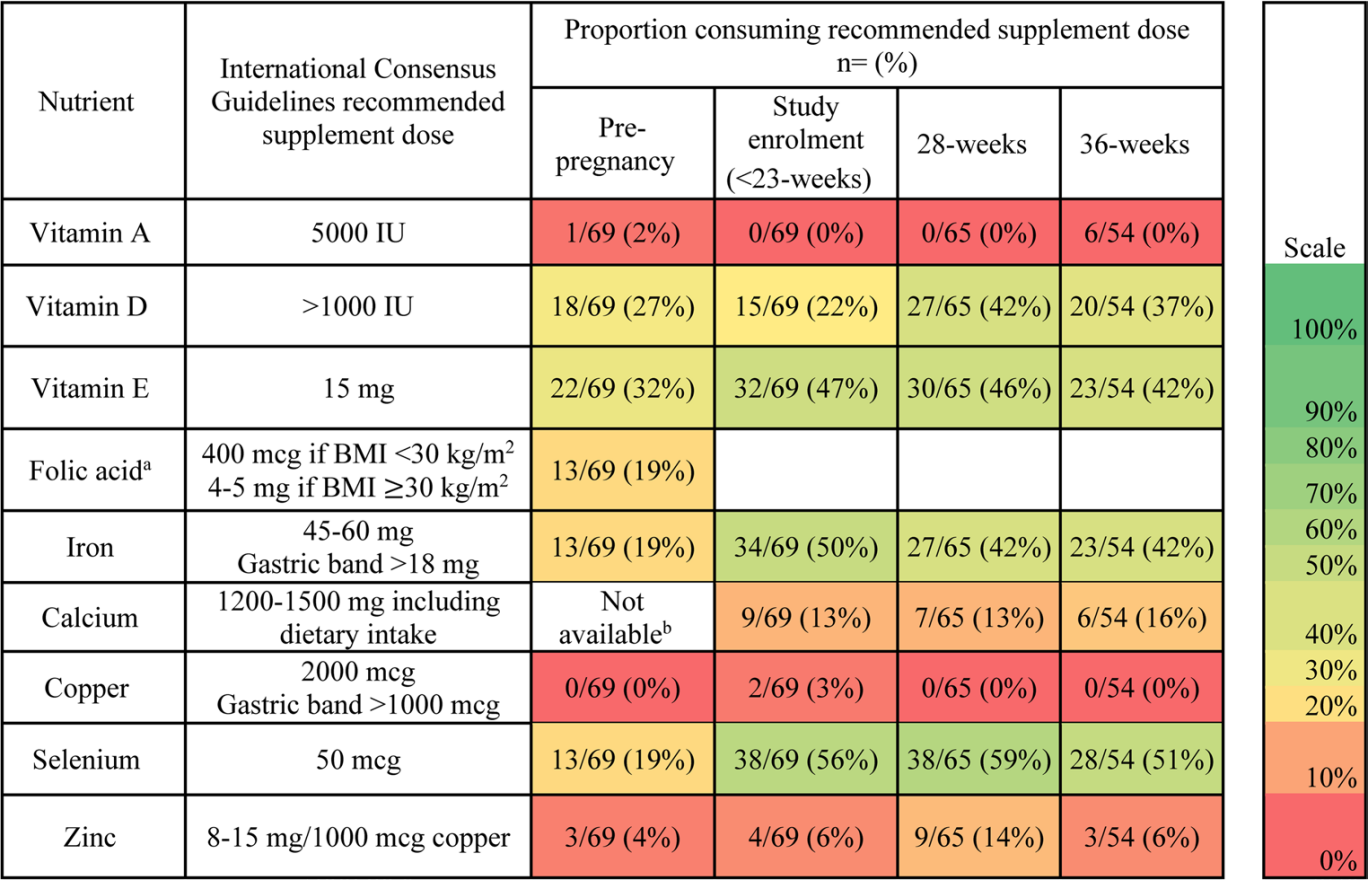
Heat map demonstrating the proportion of participants meeting the International Consensus Guidelines supplement dose recommendations prior to and throughout pregnancy following bariatric surgery. ^a^Folic acid supplements only recommended pre-pregnancy and until 12-weeks, therefore adherence cannot be reported for study recruitment, 28-weeks or 36-weeks. ^b^Dietary calcium intake not available pre-pregnancy therefore the proportion meeting the recommended dose pre-pregnancy cannot be determined. Abbreviations: International Units (IU), milligrams (mg), micrograms (mcg), Body Mass Index (BMI), kilograms per meters square (kg/m^2^)

In 2019, Shawe et al. [7] released International Consensus Guidelines for the care of pregnant women with a history of bariatric surgery, recommending women to take a multivitamin multimineral supplement (minimum micronutrient doses provided in Fig. 1) alongside biochemical monitoring of vitamin and mineral status prior to and throughout pregnancy. These recommendations were made based on expert opinion and a small number of non-randomized studies, many of which had methodological limitations. The incidence of micronutrient deficiency in an Australian cohort of pregnant women after bariatric surgery is yet to be evaluated and the efficacy of recommended supplementation is unknown. This study aimed to assess the proportion of women meeting recommended supplement doses during pregnancy following bariatric surgery. A secondary aim was to examine the influence of micronutrient intake (from diet and supplementation) on the development of micronutrient deficiency in an Australian cohort of pregnant women with history of bariatric surgery. This study is nested within a prospective multicenter cohort study examining maternal nutrition during pregnancy following bariatric surgery [21, 22].

## Methods

### Study Design

This prospective observational study was conducted across one quaternary and three secondary referral hospitals in Australia (June 2022 - February 2024). The study received ethical approval (Metro North Hospital and Health Service Human Research and Ethics Committee EC00168) and was registered (Australia and New Zealand Clinical Trials Registry registration no. ACTRN12623000495628 null).

### Study Population

Women aged 18–45 years with a singleton pregnancy < 23 weeks’ gestation and a history of bariatric surgery (gastric band, gastric sleeve, gastric bypass or revisional procedure) were eligible. Exclusion criteria included twins or triplet pregnancy, conditions affecting micronutrient status other than bariatric surgery (such as inflammatory bowel disease), type one diabetes mellitus, or women who were unable to participate in English. Eligible women were approached by healthcare providers and, if interested, contacted by the lead researcher to confirm eligibility, and provide electronic consent using REDCap. Women who participated in the study entered a draw for one of five AUD $20 grocery vouchers. No other incentive was provided to participants.

### Data Collection

Participants completed online surveys at enrolment (< 23 weeks), 28- and 36-weeks’ gestation reporting demographics, bariatric surgery procedure type and date, anthropometry (height, pre-operative, and pre-pregnancy weight). Supplement use was self-reported for the preconception period and two weeks prior to completing each survey; including the name, brand, and dose of all supplements (multivitamins and single nutrient supplements). Adherence was assessed by quantifying the number of missed doses each week using the Brief Medication Adherence Questionnaire [23]. Dietary intake was assessed using three non-consecutive multiple pass 24-hour diet recalls collected at study enrolment, 28-weeks and 36-weeks gestation, resulting in up to nine diet recalls per participant. Diet recalls were collected via telephone by the lead researcher and entered into the ASA24-Australia, which uses the Australian Food, Supplement, and Nutrient Database 2011–2013, as reported previously [22]. Dietary recall method was used because alternative methods, such as food frequency questionnaires, are not validated for use in populations with bariatric surgery and rely on standardized portion sizes that may not accurately reflect post-surgical eating patterns. Pregnancy and biochemical data (gravida, parity, haemoglobin, ferritin, serum retinol, total B12, 25-hydroxy-vitamin D, alpha-tocopherol, copper, zinc and selenium) were collected during routine clinical practice and extracted from medical records after birth. Records also identified access to dietitian advice, administration of intramuscular vitamin B12 replacement and iron infusions. Clinical care was guided by existing clinical guidelines at each site and no changes were made for study participants [7, 24].

### Data Analysis

Average daily supplement dose was calculated by multiplying the nutrient content of supplements by the number of tablets taken per week, subtracting missed doses then dividing by seven. Supplement intake was dichotomised as ‘meeting’ or ‘not meeting’ the International Consensus Guidelines [7] minimum recommended dose of each micronutrient (Fig. 1). Dietary micronutrient intake is described as average intake over 24 h from all dietary recalls completed. To determine whether participants met the recommended intake of calcium, dose from supplements was combined with daily average calcium dietary intake to account for both dietary and supplementary sources. Biochemical micronutrient deficiency was determined as any measurement below the reference range provided in Table 1. The identification of micronutrient deficiency was made based on the best available evidence and a rationale for each threshold is provided in Supplementary materials (Table S1). Pregnancy-onset deficiency was categorised using the following criteria:

**Table 1:** Prevalence of micronutrient deficiency during pregnancy following bariatric surgery. The prevalence of anaemia, vitamins A, B12, D, E, folate, iron, selenium, copper and zinc deficiency during pregnancy following bariatric surgery it stated below from original data as well as imputed data

Micronutrient	Criteria for deficiency	Original data		Imputed data
		Proportion with deficiency *n*= (%)	Proportion missing data *n*= (%)	Proportion with deficiency *n*= (%)
Vitamin A	Serum retinol <1.05 mmol/L [25]	8/69 (12%)	25/69 (36%)	21/69 (30%)
Vitamin B12	Total B12 <150 pmol/L [26]	26/69 (38%)	21/69 (30%)	38/69 (54%)
Vitamin D	25-OH <50 nmol/L [27]	12/69 (17%)	16/69 (23%)	21/69 (30%)
Vitamin E	Trimester 1 <16.25 μmol/LTrimester 2 <19.74 μmol/L Trimester 3 <21.83 μmol/L [28]	0/69 (0%)	49/69 (71%)	Not applicable
Folate	Trimester 1 <5.8 nmol/LTrimester 2 <1.8 nmol/LTrimester 3 <3.2 nmol/L [29]	0/69 (0%)	20/69 (29%)	0/69 (0%)
Iron	Ferritin <30 ng/mL [30]	43/69 (62%)	13/69 (19%)	49/69 (72%)
Anaemia	Haemoglobin <110 g/L or <105 g/L in trimester 2 [31]	21/69 (30%)	3/69 (4%)	Not applicable
Iron deficiency anaemia	Iron deficiency anaemia: Ferritin <30 ng/mL and haemoglobin <110 g/d or <105 g/L in trimester 2 [30]	15/69 (22%)	11/69 (16%)	20/69 (29%)
Selenium	Trimester 1 <18 μmol/LTrimester 2 <26 μmol/L Trimester 3 <21 μmol/L [29]	3/69 (4%)	55/69 (80%)	Not applicable
Zinc	Trimester 1 <1.47 μmol/L Trimester 2 <0.95 μmol/LTrimester 3 <0.9 μmol/L [29]	6/69 (9%)	37/69 (54%)	Not applicable
Copper	Trimester 1 <8.5 μmol/LTrimester 2 and 3 <7.7 μmol/L [32]	2/69 (3%)	55/69 (80%)	Not applicable
Overall	Proportion with at least one micronutrient deficiency during pregnancy	56/69 (81%)	3/69 (4%)	Not applicable

Abbreviations: millimoles per litre (mmol/L), picomoles per litre (pmol/L), nanomoles per litre (nmol/L), micromoles per litre (μmol/L), grams per litre (g/L), nanograms per millilitre (ng/mL)

- Pregnancy-onset anaemia: normal haemoglobin levels before 12 weeks’ gestation but deficient thereafter.
- Pregnancy-onset vitamin A, B12, D, iron or zinc deficiency: normal serum concentration before 20 weeks’ gestation but deficient thereafter.

Analysis was performed using the Statistical Package for the Social Sciences (version 30, 2024). The distribution of continuous variables was assessed using the Shapiro Wilk test, which informed the use of mean ± standard deviation (SD) or median and interquartile range (Q1-Q3) to describe normally and non-normally distributed variables, respectively. Change in supplement dose across pregnancy was assessed with a repeated measures ANOVA or Friedman’s test. Independent samples t-tests and Mann-Whitney U tests were used to compare micronutrient intake between participants with and without deficiency and between those who did and those who did not receive advice from a dietitian. Spearman’s correlation examined the relationship between micronutrient consumption from dietary sources and supplements with maternal micronutrient blood concentrations each trimester. Univariate binary logistic regression examined the impact of micronutrient intake (supplement dose at each timepoint, or dietary intake) on the odds of developing vitamins A and D deficiency. Multivariate binary logistic regression examined predictors of vitamin B12 and iron deficiency. Models included dietary intake, mean supplement dose and parenteral replacement (intramuscular vitamin B12 or iron infusions, respectively). Regression models used the enter method and were examined for multicollinearity (variance inflation factor and tolerance), outliers (Mahalanobis distance), and linearity (log transformation in logistic regression). A post-hoc power analysis was conducted for the multivariate models using the Nagelkerke R^2^ and the p-value. A p-value of < 0.05 was considered statistically significant. Odds ratios were reported alongside the 95% confidence intervals (OR (95% CI)).

### Management of Missing Data

Micronutrient testing practices varied between recruiting facilities, leading to missing micronutrient data. Management of missing data were conducted in accordance with the recommendations published by Jakobsen et al. [33]. As more than 40% of participants had missing zinc, vitamin E, selenium and copper levels, data imputation was deemed inappropriate and analysis for these variables used pairwise deletion. For variables with 5–40% missing data (vitamins A, B12, D, folate, iron deficiency and iron deficiency anemia), the pattern of missingness was examined to inform data imputation methods. Little’s MCAR test, conducted with facility, model of care, ethnicity, surgery type, language and education level as explanatory variables, was statistically significant [34] (*X*^*2*^*=* 1515.35, *p* = 0.000). This informed the decision to perform multiple imputation using predictive mean matching with 10 iterations, using surgery type, ethnicity, model of antenatal care and parity as predictors [34]. As missingness ranged between 16 and 36% across imputed variables, 40 imputations were performed (35). Results from imputed datasets were pooled using Rubin’s rules and reported alongside analysis from the original data.

## Results

**Fig. 2:**
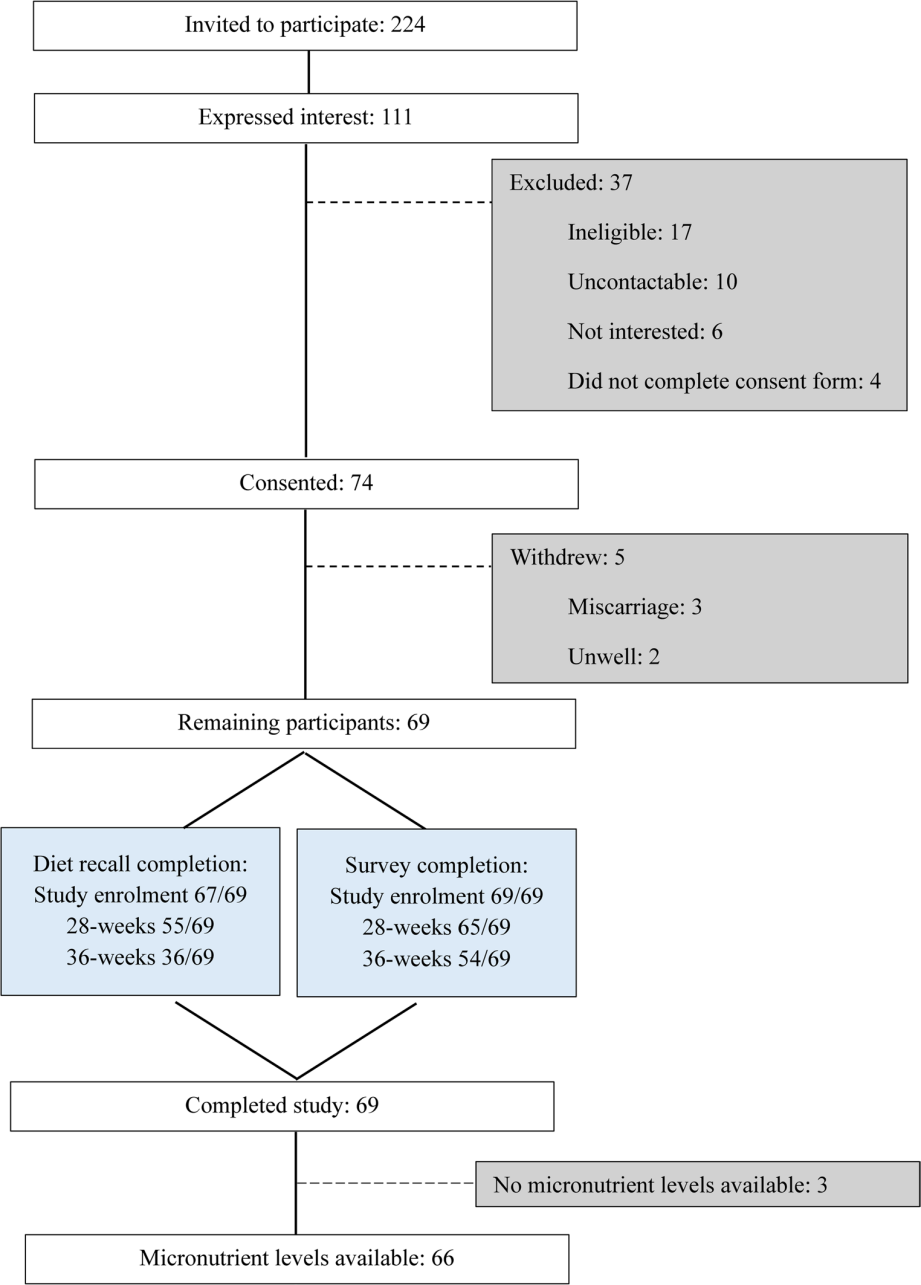
Study recruitment and completion rate

The study included 69 participants, of whom 66 had micronutrient monitoring conducted during pregnancy (Fig. 2). Sixty-eight percent (47/69) of participants had received a sleeve gastrectomy with a median surgery-to-conception interval of 30 (14–65) months (Table 2). Participants completed a median of 6 (5–9) dietary recalls across the pregnancy (range 1–9). Recall completion by gestational stage is provided in Supplementary material, Table S2).

**Table 2:** Participant demographics and care provided during pregnancy. The characteristics of study participants are stated below

	Median (Q1-Q3) or *n*= (%)	Missing data *n*= (%)
Maternal age (years)	32±4.8^a^	0/69 (0%)
Parity	1 (0–2)	1/69 (1%)
Nulliparous, *n*= (%)	25/69 (36%)	
Bariatric surgery procedure, *n*= (%)		0/69 (0%)
Gastric band	1/69 (2%)	
Sleeve gastrectomy	47/69 (68%)	
Gastric bypass	21/69 (30%)	
Revisional surgery, *n*= (%)	5/69 (7%)	1/69 (1%)
Bariatric surgery-to-conception interval, (months)	29 (14–65) Range: 0-186	1/69 (1%)
BMI before bariatric surgery, (kg/m^2^)	45.1 (40.4–53.1) Range: 32.7–73.1	1/69 (1%)
BMI pre-pregnancy, (kg/m^2^)	31.7 (27.0-34.3) Range: 18.4–50.4	3/69 (4%)
Ethnicity, *n*= (%)		1/69 (1%)
Caucasian	53/69 (77%)	
Aboriginal and/or Torres Strait Islander	7/69 (10%)	
Pacific Islander	2/69 (3%)	
Other ethnicity	6/69 (9%)	
Education completed, *n*= (%)		1/69 (1%)
Postgraduate or undergraduate degree	16/69 (23%)	
Trade, technical certificate or diploma	27/69 (39%)	
Completed schooling	19/69 (28%)	
Did not complete schooling	6/69 (9%)	
Antenatal model of care received, *n*= (%)		3/69 (4%)
General Practitioner shared care	14/69 (20%)	
Obstetric led care	17/69 (25%)	
Midwife led care (includes midwifery group practice)	34/69 (49%)	
Other	1/69 (1%)	
Attended dietitian appointments during pregnancy, *n*= (%)	59/69 (86%)	2/69 (3%)
Gestation of first appointment, (weeks)	17 (15–20)	
Number of appointments attended during pregnancy	4 (3–5)	

^a^Mean±SD

Abbreviations: *BMI* Body Mass Index, *kg/m*^*2*^ kilograms per meters square, *SD* standard deviation

### Use of Micronutrient Supplements Throughout Pregnancy

Multivitamin use ranged from 38/69 (55%) pre-pregnancy, to 55/69 (80%) at enrolment then remained stable at 50/65 (77%) at 28-weeks and 41/54 (75%) at 36-weeks. The median number of missed doses was low for all supplements, but was higher pre-pregnancy compared to during pregnancy (supplementary material, Table S3). Participants met the International Consensus Guidelines supplement recommendations most often for selenium (59%, 38/65 meeting recommended dose at 28-weeks) and least often for vitamin A (0%, 0/68 meeting recommended dose during pregnancy, Fig. 1). The dose of vitamin A (*p* = 0.026), iron (*p* = < 0.001), selenium (*p* = < 0.001) and zinc (*p* = < 0.001) supplements increased during pregnancy, whereas folic acid decreased (p = < 0.001). Supplement doses of vitamin B12, D, E, calcium and copper were unchanged throughout pregnancy (*p* > 0.05, Supplementary material, Table S4).

Most participants (59/69, 85%) received advice from a dietitian. This was associated with an increase dose of calcium (28-weeks 125 (2-325) mg vs. 0 (0–0) mg, *p* = 0.005; 36-weeks 104 (0-286) mg vs. 0 (0–59) mg, *p* = 0.009), vitamin E (28-weeks 19 (0–62) mg vs. 0 (0–0) mg *p* = 0.008; 36-weeks 14 (0–55) mg vs. 0 (0–4) mg, *p* = 0.009) and copper (28-weeks 606 (0-1000) mcg vs. 0 (0–0) mcg, *p* = 0.046) and a reduction in the dose of folic acid supplementation (36-weeks 572 (277–800) mcg vs. 375 (0-475) mcg, *p* = 0.042). Advice from a dietitian did not significantly change vitamin A, D, iron, or zinc supplement dose at any timepoint (*p* > 0.05).

### Micronutrient Deficiency During Pregnancy

Maternal serum micronutrient levels each trimester are reported in Table 3. Serum folate concentration in the second and third trimesters were positively correlated with the dose of folic acid supplement at 28- (second trimester *r* = 0.461, *p* = 0.15; third trimester *r* = 0.703, p = < 0.001) and 36-weeks (second trimester *r* = 0.542, *p* = 0.008; third trimester *r* = 0.599, *p* = 0.003). Serum vitamin D concentration during the first trimester was negatively correlated with supplement dose at 28- (*r*=-0.414, *p* = 0.028) and 36-weeks (*r*=-0.479, *p* = 0.021). In the second trimester, vitamin D levels were significantly but modestly correlated with supplement dose at 28-weeks (*r* = 0.370, *p* = 0.022). Third trimester vitamin D levels were also correlated with vitamin D supplement dose at 28-weeks (*r* = 0.419, *p* = 0.017). Serum vitamin E was correlated with dietary vitamin E intake (*r* = 0.462, *p* = 0.041). Serum selenium was strongly and significantly correlated with selenium supplement dose at study enrolment (*r* = 0.859, p = < 0.001). Serum zinc in the second trimester was negatively correlated with dietary zinc intake (*r*=-0.455, *p* = 0.038), but positively correlated with zinc supplementation pre-pregnancy (*r* = 0.516, *p* = 0.017). There was no significant correlation between vitamin A intake and retinol concentrations, vitamin B12 intake and total B12 concentration. Additionally, there were no significant correlations between ferritin or haemoglobin concentration and consumption of iron, B12, or folic acid from dietary sources or supplements (*p* > 0.05).

**Table 3:** Maternal serum micronutrient concentration throughout pregnancy after bariatric surgery. This table describes the mean maternal blood concentrations of micronutrients during each trimester of pregnancy

Nutrient	Unit	Maternal serum concentration								
		First trimester			Second trimester			Third trimester		
		Mean±SD	Range	Proportion of missing datan= (%)	Mean±SD	Range	Proportion of missing datan= (%)	Mean±SD	Range	Proportion of missing datan= (%)
Haemoglobin	g/L	128±9.2	100-147	34/69 (49%)	116±9.2	98-141	24/69 (35%)	114±14.5	85-142	6/69 (9%)
Ferritin	ng/mL	45 (25-82)^a^	8-162	46/69 (67%)	38 (69-72)^a^	2-703	30/69 (44%)	11 (7-29)^a^	2-853	23/46 (33%)
Vitamin A (serum retinol)	μmol/L	1.26±0.36	0.7-1.8	60/69 (87%)	1.53±0.29	1.0-2.2	39/69 (56%)	1.24±0.28	0.7-1.9	45/69 (65%)
Vitamin B12 (total serum)	pmol/L	262 (169-339)^a^	57-560	49/69 (71%)	187 (122-298)^a^	38-1476	39/69 (56%)	150 (113-191)^a^	29-930	40/69 (58%)
Vitamin D (25-hydroxy vitamin D)	nmol/L	63±15.7	29-93	40/69 (58%)	70 (61.3-82)^a^	41-146	29/69 (42%)	85±30.0	41-172	36/69 (52%)
Vitamin E (alpha-tocopherol)^b^	μmol/L	35.8±7.9	20.0-52.0	49/69 (71%)						
Folate (serum folate)	nmol/L	32.1±12.7	7.7-54.2	52/69 (75%)	36.8±12.7	12.0-53.1	41/69 (59%)	36.8±11.1	4.7-51.6	46/69 (67%)
Selenium (serum)^c^	μmol/L	1.14±0.16	0.88-1.50	49/69 (71%)						
Copper (serum)^d^	μmol/L	32±9.4	12-45	55/69 (80%)						
Zinc (serum)	μmol/L	1 (1-8.5)^a^	1-9	64/69 (93%)	8 (3-9)^a^	1-9	48/69 (70%)	7 (4-8)^a^	1-9	52/69 (75%)

^a^Median (Q1-Q3)

^b^Insufficient data to report mean per trimester, serum vitamin E taken at 17 (23)^I^ weeks

^c^Insufficient data to report mean per trimester, serum selenium taken at 20±9 weeks

^d^Insufficient data to report mean per trimester serum copper taken at 21±8 weeks

Abbreviations: mmol/L millimoles per litre, pmol/L picomoles per litre, nmol/L nanomoles per litre, μmol/L micromoles per litre, g/L grams per litre, ng/mL nanograms per millilitre, SD standard deviation

Eighty-five percent of participants (56/66) were found to have biochemical evidence of a micronutrient deficiency. Iron and vitamin B12 deficiency were most common, affecting 49/69 (72%) and 38/69 (54%) of participants, respectively (Table 1). Most cases of anemia (82%, 14/17) were categorized as pregnancy-onset, as they developed after 12 weeks’ gestation. Similarly, most vitamin A (83%, 5/6), vitamin B12 (66%, 8/12), and zinc deficiencies (100%, 2/2) developed after 20 weeks’ gestation, whereas only 3/9 (33%) cases of vitamin D and 7/18 (39%) of iron deficiencies developed after this timepoint. However, sample sizes for identifying pregnancy-onset deficiency were small across all nutrients (*n* = 2–18).

Micronutrient intake from supplements and dietary sources were similar between participants with and without micronutrient deficiency using both original and imputed data (*p* > 0.05), apart from vitamin B12. Oral B12 supplement dose at 36-weeks were lower in women with a B12 deficiency than those without in the original dataset (2.6 (34.8) mcg vs. 39.2 (395.2) mcg, *p* = 0.025). This was consistent in 8 out of the 40 imputed datasets (*p* = 0.005–0.039), however most (32/40) imputations were non-significant (*p* > 0.05). Intramuscular B12 replacement was received by 22% (15/69) participants, of whom 60% (9/12) had evidence of B12 deficiency. Iron infusions were received by 30% (15/67) of participants. Univariate regression analysis was unable to identify predictors of vitamin A or vitamin D deficiency (supplementary material, Table S5 and S6). Multivariate analysis was also unable to identify significant predictors of vitamin B12 (X^2^ = 4.125, *p* = 0.245) or iron deficiency (X^2^ = 1.938, *p* = 0.585). Power analysis revealed that both models had limited statistical power (5–63% and 5–61%, respectively). The OR(95% CI) are provided in supplementary material (Table S7 and S8).

## Discussion

This study examined whether women attending public hospital antenatal care met the recommended micronutrient supplementation during pregnancy following bariatric surgery. Although most participants used a multivitamin before and during pregnancy, few achieved the doses recommended in the International Consensus Guidelines and 85% developed a micronutrient deficiency, most commonly iron and vitamin B12. Micronutrient intake influenced some biochemical serum levels, however our analysis did not detect a significant impact on the development of deficiency during pregnancy. This suggests that women who conceive after bariatric surgery are at significant risk of iron and B12 deficiency and this likely has a complex aetiology beyond simply dietary intake and supplement use. 

This is the first prospective study to describe micronutrient supplement dose and adherence among pregnant bariatric surgery recipients over the course of their pregnancies. Consistent with our findings, a retrospective study reported less than half of pregnant women met recommended supplement doses during pregnancy post-bariatric surgery [36]. One barrier may be the limited availability of multivitamin formulations that meet the recommended micronutrient doses. For example, vitamin A is frequently omitted from pregnancy multivitamins due to concerns of teratogenicity associated with retinol doses exceeding 10,000 IU, which is the upper limit for intake in pregnancy in Australia [37, 38]. The generalizability of this literature to bariatric surgery recipients is uncertain as they may have reduced dietary intake of vitamin A and fat, which is required for its absorption. Bypass procedures further limit chyle contact with the jejunum, the primary site of vitamin A absorption [13]. Existing guidelines recommend women receive 5000 IU of vitamin A during pregnancy exclusively from beta-carotene, which has not been associated with teratogenicity [37–39]. However, beta-carotene has suboptimal bioavailability [40], particularly among individuals who are already vitamin A deficient [41], and supplements containing sufficient doses of beta-carotene are not available in Australia. Fear of adverse effects has been cited by consumers who were reluctant to use vitamin A containing supplements during pregnancy [21]. The lack of clarity in existing guidelines may also contribute to consumers receiving conflicting advice regarding supplement recommendations in pregnancy. Together, these factors may explain why none of our participants met the recommended doses of vitamin A. Although bariatric surgery-specific supplements exist, they are not specifically designed or marketed for pregnancy and evidence for their efficacy in pregnancy is limited [42]. Consumers also report barriers such as gastrointestinal side effects, cost, pill burden and receiving conflicting advice from health professionals [21, 43]. Our findings suggest tailored dietary advice during pregnancy can improve supplement intake, as women who had at least one appointment with a dietitian increased their micronutrient consumption. Receiving individualised nutrition care also aligns with consumer preferences for their antenatal care [21]. Yet, prior research suggests many health professionals feel underprepared to provide such advice, citing a lack of knowledge, time and multidisciplinary collaboration as barriers [44]. The limited proportion of women meeting recommended supplementation in our cohort likely reflects both these systemic issues and the personal challenges women face in sustaining supplement regimens. This study uniquely assessed micronutrient intake considering both dietary sources and supplement use considering missed doses. While micronutrient intake influenced serum concentrations of some nutrients, this relationship was not consistent across all nutrients examined. Intake of iron, folate, and vitamin B12 was not correlated with serum ferritin or haemoglobin concentrations. This likely reflects several physiological and methodological factors. In this cohort, which consisted predominantly of sleeve gastrectomy recipients, absorption of both iron and vitamin B12 is disrupted by reduced gastric acid secretion [45]. Iron absorption is mediated by several complex interdependent factors such as competitive inhibition and iron status, which were not captured in this analysis. In addition, serum ferritin and haemoglobin are relatively insensitive biomarkers of short-term dietary intake, and supplementation may take 3 to 6 months to normalize serum concentrations in non-pregnant populations [46]. Contrastingly, serum measures of selenium and folate are more responsive to recent intake [47]. This may explain why correlations between intake and serum levels were more readily observed for these nutrients. The mechanism for the negative correlation between dietary zinc intake and zinc concentration is not clear but may reflect the limited sample size available for this analyses. 

Deficiencies detected early in pregnancy, such as iron and vitamin D, likely indicate pre-existing low or negligible stores of these micronutrients or an inability to mobilise them effectively [48]. Given that less than one third of participants met supplement recommendations preconception for iron and vitamin D, inadequate supplementation may contribute to this finding. Other deficiencies (vitamin A, B12 and zinc) appeared to develop during pregnancy. This may reflect the choice to use thresholds for vitamin A and B12 deficiency that do not account for pregnancy-induced haemodilution. However, previous research has also been unable to establish a clear relationship between supplement use and deficiency risk during pregnancy [42, 49, 50]. Only one-fifth of participants in this study developed vitamin A deficiency compared to previous studies reporting one-third [49] to two-thirds becoming deficient (< 1.05 $\mu$mol/L [42, 50] and < 1.22 $\mu$mol/L [49]) despite consuming larger doses of vitamin A (4000–5000 IU compared to 0-555 IU in this study) [42, 51]. Similarly, other studies of women using larger doses of vitamin D, E, selenium, zinc and folate experience similar rates of deficiency to women in this study [49, 50, 52]. These previous studies did not consider adherence to supplementation when reporting overall supplement dose, potentially leading to over estimation of mean supplement intake compared to our study. Iron and B12 deficiency was more common in our cohort than previously reported [42, 50, 53]. However, comparisons across studies are complicated by substantial heterogeneity in the diagnostic thresholds used to define deficiency. For example, B12 deficiency has been defined as < 87 pmol/L, leading to a 0–4% rate of deficiency [53] whereas when defined as < 200 pmol/L, 44% of pregnant bariatric-surgery recipients develop deficiency [42]. The reference ranges used to diagnose micronutrient deficiency are poorly validated in pregnancy [13, 28, 29]. A complex interplay of factors, such as preconception nutrient status, gestational changes in metabolism [54], supplement bioavailability, genetics, the presence of chronic or acute inflammation, bariatric surgery procedure, individual anatomy and gut adaptation post-operatively may all contribute to the development of deficiency [55]. This highlights the limitations of considering micronutrient intake and deficiency risk in isolation. Future research should therefore move beyond micronutrient intake alone to consider the broader physiological and surgical influences to better predict micronutrient deficiency risk during pregnancy following bariatric surgery and develop individualised treatment protocols focussed on improving long term health outcomes.

Micronutrient deficiency in pregnancy can adversely affect perinatal outcomes [43, 48, 56]. Bariatric surgery recipients have difficulty meeting the micronutrient demands of pregnancy from dietary sources alone and may have impaired micronutrient absorption [1, 22]. However, multivitamin use is not without risks – for example vitamin B6 toxicity arising from long term multivitamin use has occurred in bariatric surgery recipients [56] and elevated B6 levels in pregnant surgery recipients have been observed [42]. Though some degree of supplementation appears necessary to address gaps in dietary intake, this study was unable to demonstrate the efficacy of the doses recommended in the International Consensus Guidelines. Only half of participants reported preconception multivitamin use, with adherence lowest during this period, indicating that many women may enter pregnancy with depleted or sub-optimal micronutrient reserves. These findings support the clinical value of promoting preconception multivitamin supplementation, even when ideal formulations or dosing strategies have yet to be established. Supporting women before conception also enhances opportunities to address dietary patterns that may lead to gaps in micronutrient intake [22]. Given the high rates of iron and B12 deficiency, monitoring these specially may warrant greater priority than routine surveillance of less commonly affected micronutrients (vitamins E, folate, selenium, copper and zinc) particularly among gastric sleeve recipients using multivitamins. As many deficiencies were present early in pregnancy, screening for deficiency preconception or in early pregnancy is likely to be most beneficial and inform more tailored supplement regimens. More research is needed to identify predictors of deficiency to allow for risk-stratified monitoring, ensuring comprehensive but cost-effective antenatal care.

Strengths of this study include its prospective design, limiting recall bias, and the use of validated methods to assess dietary intake and supplement adherence [23]. However, several limitations should be acknowledged. Although up to nine 24-hour dietary recalls were collected across pregnancy, this may not have fully captured habitual intake, or seasonal variation food patterns and therefore micronutrient intake [57]. The reliance on self-report introduces recall and social desirability bias, potentially leading to misreporting of dietary intake and over-estimation supplement adherence. Supplement adherence was assessed using a validated questionnaire originally developed for chronic disease medications, but this tool has not been specifically validated for nutritional supplements in this population, and objective measures of supplement adherence were not collected. Biochemical data were obtained from routine clinical care rather than standardized research sampling, meaning timing, fasting status, seasonality, and assay variability were not controlled. Additionally, variations in clinical practice produced large proportions of missing biochemical data, limiting statistical power. This may have also introduced bias if testing was more likely in symptomatic women. Consequently, null findings should be interpreted as an inability to detect associations rather than evidence of no effect. Deficiencies were defined using the best available thresholds informed by pregnancy and bariatric surgery literature; however, these cut-offs are sub-optimally defined and vary between laboratories [29]. Although the diagnosis of anaemia, vitamin E, copper, zinc, folate, and selenium deficiencies were adjusted for haemodilution, this may have impacted the reporting of iron, vitamins A, B12 and D deficiency, possibly leading to over-reporting of deficiency rates. Several other factors may confound interpretation of serum micronutrient levels, such as systemic inflammation [58]. As most participants had undergone sleeve gastrectomy, generalizability to gastric bypass or revisional procedures is limited. This cohort may be at greater risk of micronutrient deficiency due to greater malabsorption and poorer micronutrient intake [13, 22]. Clinical symptoms of specific micronutrient deficiency, such as night blindness, were not assessed, as the study enrolled free living community volunteers. Finally, the absence of a non-bariatric comparison group limits causal inference, as observed deficiencies may reflect the combined effects of prior obesity, pregnancy physiology, and surgical history which further complicates the examination of the relationship between micronutrient intake and deficiency [59]. Despite these limitations and confounders, this study makes an important contribution to the literature by providing real-world insights into micronutrient supplementation and status for the increasing number of women achieving pregnancy after surgical procedures for weight loss.

## Conclusion

This prospective study demonstrates that, despite frequent supplement use during pregnancy, most women did not meet guideline-recommended supplement doses following bariatric surgery, and only around half reported using a multivitamin preconception. Although micronutrient supplementation during pregnancy influenced serum concentrations of some nutrients, this study was unable to demonstrate a complete protective effect against biochemical deficiency. Supplement doses generally increased as gestation progressed; however, these findings suggest that many women may be entering pregnancy with depleted micronutrient stores, limiting the capacity of supplementation or dietary interventions during pregnancy to correct deficiencies. Persistently high rates of iron and vitamin B12 deficiency highlight the importance of targeted micronutrient screening early in pregnancy. Together, these findings underscore the limitations of current one-size-fits-all supplementation recommendations and emphasize the importance of optimizing micronutrient status prior to conception. Individualized supplementation strategies that account for nutrient status, bariatric procedure, and dietary intake are likely required. Our study suggests that dietitians are well positioned to deliver this care so ensuring timely access to dietetic support should be a priority for healthcare services.

## Electronic Supplementary Material

Below is the link to the electronic supplementary material.

Supplementary Material 1

## Data Availability

The data that support the findings of this study are not openly available due to reasons of sensitivity and are available from the corresponding author upon reasonable request. Data are located in controlled access data storage at The University of Queensland secure online database.
