# Supplementary material for: Micronutrient Deficiency During Pregnancy After Bariatric Surgery: The Role of Micronutrient Supplements and Dietary Intake
Source: Obes Surg. 2026 Mar 25;36(5):2191–203. doi: 10.1007/s11695-026-08576-7 (PMC13222237; doi:10.1007/s11695-026-08576-7)
Supplement: Supplementary file 1 — Supplementary Material 1 [file 11695_2026_8576_MOESM1_ESM.pdf]

Supplementary Material

Table S1: Rationale for the thresholds used to define micronutrient deficiency during pregnancy following bariatric surgery

This table provides a brief rationale for the thresholds chosen to identify biochemical deficiency during pregnancy following bariatric surgery considering the impact of pregnancy-related haemodilution and the influence on perinatal health outcomes.

Micronutrient	Threshold for deficiency	Summary of evidence supporting this criteria
Anaemia	Haemoglobin < 110 g/L or < 105 g/L in the second trimester	World Health Organization criteria used internationally in to identify anaemia in pregnancy (56, 60, 61).
Iron deficiency	Ferritin < 30 ug/L	Used in clinical practice to identify risk of iron deficiency anaemia (56, 60, 61).
Vitamin A deficiency	WHO criteria for adults: serum retinol < 1.05 μmol/L	World Health Organization criteria used to identify vitamin A deficiency in population studies (54), supported by a large study in pregnancy (62). Used by majority of previous studies of pregnant bariatric surgery recipients (42, 43, 63–66).
Vitamin B12 deficiency	WHO criteria for adults: total B12 < 150 pmol/L	Adult diagnostic criteria supported by one large meta-analysis of pregnant women (17).
Vitamin D deficiency	Institute of Medicine criteria: 25-hydroxy-vitamin D < 50 nmol/L	Extensively applied in the literature, correlated with preterm birth in a meta-analysis (67), aligns with previous studies of bariatric surgery recipients (42, 45, 68–70).
Vitamin E deficiency	Serum alpha-tocopherol < 16.25 μmol/L first<br>< 19.74 μmol/L second and<br>< 21.83 μmol/L third trimester	Based on a large cross sectional large study conducted in China in the absence of other data (47).
Folate deficiency	Abbassi-Ghanavati et al. (46) criteria: serum folate < 5.8 nmol/L first,<br>< 1.8 nmol/L second and<br>< 3.2 nmol/L third trimester	Only available data in pregnancy (46).
Selenium deficiency	Abbassi-Ghanavati et al. (46) criteria:<br>Serum selenium < 1.47 μmol/L	Only available data (46) and used by previous studies of bariatric surgery recipients (44, 71).

	first,<br>< 0.95 μmol/L second and<br>< 0.9 μmol/L third trimester	
Copper deficiency	Abbassi-Ghanavati et al. (46) criteria: Serum copper < 17.6 μmol/L first,<br>< 26.0 μmol/L second and<br>< 20.5 μmol/L third trimester	Only available data (46), no previous studies on copper deficiency in pregnant bariatric surgery recipients.
Zinc deficiency	Abbassi-Ghanavati et al. (46) criteria:<br>Serum zinc < 8.5 mmol/L first,<br>< 7.8 mmol/L second and third trimester	Only available data (46) and used by select previous studies including pregnant bariatric surgery recipients (41, 65).

Abbreviations: millimoles per litre (mmol/L), picomoles per litre (pmol/L), nanomoles per litre (nmol/L), micromoles per litre (μmol/L), grams per litre (g/L), nanograms per millilitre (ng/mL).

Table S2: Diet recalls completed at each gestational stage

This table outlines the completion of 24 hour dietary recalls during pregnancy of study participants.

24hour recalls completed	Proportion of participants who completed dietary recalls		
	Enrolment<br>(< 23 weeks gestation)<br>n= (%)	28-weeks gestation<br>n= (%)	36-weeks gestation<br>n= (%)
No recalls completed	2/69 (3%)	14/69 (20%)	24/69 (35%)
1 recall	3/69 (4%)	4/69 (6%)	6/69 (9%)
2 recalls	5/69 (7%)	7/69 (10%)	3/69 (4%)
3 recalls	59/69 (86%)	44/69 (64%)	36/69 (52%)

Table S3: Micronutrient supplement use and adherence prior to and throughout pregnancy after bariatric surgery

This table describes the proportion of women using multivitamin multimineral supplements as well as individual folic acid, iron, calcium and vitamin D supplements during pregnancy following bariatric surgery. Adherence to supplementation is also reported as the median percentage of missed doses per week.

Supplement type	Pre-pregnancy		Study Enrolment (< 23-weeks)		28-weeks		36-weeks	
	Proportion using a supplement n= (%)	Percent of doses missed per week Median (Q1-Q3)	Proportion using a supplement n= (%)	Percent of doses missed per week Median (Q1-Q3)	Proportion using a supplement n= (%)	Percent of doses missed per week Median (Q1-Q3)	Proportion using a supplement n= (%)	Percent of doses missed per week Median (Q1-Q3)
Multivitamin multimineral	38/69 (55%)	7 (0–26)	55/69 (80%)	0 (0–14)	50/65 (77%)	0 (0–14)	41/54 (76%)	0 (0–19)
Folic acid	4/69 (6%)	9 ± 22[a]	17/69 (25%)	0 (0–29)	13/65 (20%)	0 (0–23)	7/54 (13%)	0 (0–0)
Iron	13/69 (20%)	14 (0–36)	20/69 (29%)	0 (0–27)	26/65 (40%)	0 (0–23)	24/54 (44%)	0 (0–14)
Calcium and/or Vitamin D	13/69 (19%)	0 (0–18)	18/69 (26%)	0 (0–18)	16/65 (25%)	0 (0–27)	11/54 (20%)	0 (0–0)

[a]Mean ± SD

Abbreviations: standard deviation (SD)

Table S4: Participant micronutrient intake compared to the International Consensus Guideline recommendations for micronutrient supplementation during pregnancy following bariatric surgery (7)

This table demonstrates the median doses of micronutrients consumed from supplementation and dietary sources during pregnancy and examines changes across pregnancy.

Micronutrient	Unit	*International Consensus Guidelines recommended dose*	Micronutrient intake from supplements Median (Q1-Q3)					Micronutrient intake from dietary sources Median (Q1-Q3)
			Pre-pregnancy	Enrolment (< 23-weeks)	28-weeks	36-weeks	p-value	
Vitamin A	IU	*5000*	0 (0-1643)	0 (0-1023)	0 (2976)	555 (0-2857)	***p = 0.026***	2082 (1577–2988)
Vitamin B12	mg	*No recommendation*	1.3 (0-37.7)	2.6 (1.9–7.7)	2.6 (0.9–40.9)	2.6 (0–44.0)	p = 0.412	3.6 (2.9–4.3)
Vitamin D	IU	*> 1000*	114 (0-1000)	200 (143–989)	857 (100–1200)	600 (29-1200)	p = 0.200	Not available
Vitamin E	mg	*15*	0.8 (0–21)	13.4 (0-18.7)	10.7 (0-37.4)	10.7 (0-35.7)	p = 0.500	8.2 (7.0-9.9)
Folic acid	mcg	*First trimester:*<br>*400 if BMI < 30 kg/m²*<br>*4 000–5 000 if BMI ≥30 kg/m²*	54 (0-500)	686 (429–899)	679 (293–829)	500 (114–800)	***p < 0.001***	365 (296–435)
Iron	mg	*45–60*<br>*Gastric band recipients > 18*	5.5 (0-27.9)	45 (9–64)	29.1 (7.3-104.4)	10.1 (1.4–112.0)	***p < 0.001***	8.6 (6.5–10.0)
Calcium	mg	*1200–1500 including dietary intake*	0 (0-125)	103 (10–125)	125 (0-293)	923 (0-143)	p = 0.192	762 (632–937)
Copper	mcg	*2000*	0 (0-429)	464 (0-1000)	429 (0-1000)	401 (0-1000)	p = 0.134	Not available

		Gastric band > 1000						
Zinc	mg	*8–15 /1000 mcg copper*	1.6 (0-7.8)	10.3 (4.1–11.0)	11.0 (10–15.0)	10.2 (0-12.8)	***p < 0.001***	8.5±2.4[a]
Selenium	mcg	*50*	10.7 (0-39.3)	50.0 (26.7–55.0)	50.0 (0-84.7)	50 (15.5–72.7)	***p < 0.001***	71.0 (57.9–76.5)

[a]Mean±SD

Abbreviations: Standard deviation (SD), International Units (IU), milligrams (mg), micrograms (mcg)

Table S5: Univariate binary regression analysis of predictors of vitamin A deficiency following bariatric surgery

This table reports the results from univariate binary regression analysis exploring predictors of vitamin A deficiency during pregnancy following bariatric surgery.

Predictors	Model overall	Original data	Pooled imputed data
		OR (95%CI), p-value	OR (95%CI), p value
Dietary vitamin A intake	X^2 =0.014, p = 0.905	1.000 (0.999–1.001), p = 0.906	1.00 (1.0–1.0,), p = 0.832
Vitamin A dose from supplements pre-pregnancy	X^2 =0.148, p = 0.701	1.00 (1.000–1.001), p = 0.695	1.00 (1.0-1.001), p = 0.701
Vitamin A dose from supplements at enrolment (< 23-weeks)	X^2 =0.161, p = 0.688	1.00 (1.00–1.001), p = 0.682	1.00 (0.999–1.001), p = 0.985
Vitamin A dose from supplements at 28-weeks	X^2 =0.035, p = 0.851	1.000 (0.999–1.000), p = 0.851	1.00 (1.0–1.0), p = 0.688
Vitamin A dose from supplements at 36-weeks	X^2 =2.379, p = 0.123	1.000 (0.999–1.000), p = 0.154	1.0 (0.999-1.00), p = 0.195

Abbreviations: Odds Ratio (OR), 95% Confidence interval (95%CI)

Table S6: Univariate binary regression analysis of predictors of vitamin D deficiency following bariatric surgery

This table reports the results from univariate binary regression analysis exploring predictors of vitamin D deficiency during pregnancy following bariatric surgery.

Predictors	Model overall	Original data	Pooled imputed data
		OR (95%CI), p-value	OR (95%CI), p value
Vitamin D dose from supplements pre-pregnancy	$X^2 = 0.572$, p = 0.449	1.000 (0.998–1.001), p = 0.472	1.00 (0.999–1.001), p = 0.579
Vitamin D dose from supplements < 23-weeks	$X^2 = 0.053$, p = 0.818	1.000 (0.999–1.001), p = 0.820	1.00 (0.999–1.001), p = 0.647
Vitamin D dose from supplements 28-weeks	$X^2 = 4.447$, p = 0.035	1.001 (1.000–1.001), p = 0.138	1.00 (1.00-1.001), p = 0.259
Vitamin D dose from supplements 36-weeks	$X^2 = 4.024$, p = 0.045	1.001 (1.000–1.002), p = 0.056	1.001 (1.00-1.001), p = 0.233

Abbreviations: Odds Ratio (OR), 95% Confidence interval (95%CI)

Table S7: Multivariate binary regression analysis of predictors of vitamin B12 deficiency during pregnancy following bariatric surgery

This table reports the results from multivariate binary regression analysis exploring predictors of vitamin B12 deficiency during pregnancy following bariatric surgery with dietary intake, B12 supplement dose and use of intramuscular B12 replacement included in the model.

Predictors	Model overall	Original data	Pooled imputed data
		OR (95%CI), p-value	OR (95%CI), p value
Dietary B12 intake	X^2=4.125, p = 0.245	0.868 (0.523–1.440), p = 0.583	0.870 (0.533–1.1419), p = 0.577
Mean B12 supplement dose		0.998 (0.993–1.002), p = 0.304	0.999 (0.994–1.003), p = 0.509
Receipt of intramuscular B12 replacement		2.766 (0.600-12.750), p = 0.192	2.103 (0.480–9.207), p = 0.323

Abbreviations: Odds Ratio (OR), 95% Confidence interval (95%CI)

Table S8: Multivariate binary regression analysis of predictors of iron deficiency during pregnancy following bariatric surgery

This table reports the results from multivariate binary regression analysis exploring predictors of iron deficiency during pregnancy following bariatric surgery with dietary intake, iron supplement dose and use of iron infusions were included in the model.

Predictors	Model overall	Original data	Pooled imputed data
		OR (95%CI), p-value	OR (95%CI), p value
Dietary iron intake	X^2=1.938, p = 0.585	0.937 (0.720–1.220), p = 0.629	0.988 (0.769–1.269), p = 0.924
Mean iron supplement dose		0.995 (0.981–1.010), p = 0.520	0.996 (0.982–1.010), p = 0.595
Receipt of iron infusions during pregnancy		2.290 (0.433–12.124), p = 0.330	1.973 (0.321–32.154), p = 0.320

Abbreviations: Odds Ratio (OR), 95% Confidence interval (95%CI)
